# Artificial intelligence and machine learning in bariatric surgery: a comprehensive systematic review

**DOI:** 10.1007/s00423-026-04042-1

**Published:** 2026-04-01

**Authors:** Antonio Vitiello, Giovanna Berardi, Maria Spagnuolo, Roberto Peltrini, Vincenzo Pilone

**Affiliations:** 1 Advanced Biomedical Sciences Department, Naples “Federico II” University, AOU “Federico II” – Via S. Pansini 5, Naples, 80131 Italy; 2 Attending Surgeon, University Hospital of Naples “Federico II” –, Via S. Pansini 5, Naples, 80131 Italy; 3 University of Naples “Federico II” –, Via S. Pansini 5, Naples, 80131 Italy; 4 Public Health Department, Naples “Federico II” University, AOU “Federico II” – Via S. Pansini 5, Naples, 80131 Italy

**Keywords:** Artificial intelligence, Machine learning, Bariatric surgery, Predictive modeling, Surgical education

## Abstract

**Background:**

Artificial intelligence (AI) and machine learning (ML) are increasingly integrated into metabolic and bariatric surgery (MBS), offering opportunities to enhance decision-making, optimize perioperative care, and personalize outcomes. However, their clinical adoption requires a comprehensive understanding of current applications and limitations.

**Objectives:**

To systematically review the use of AI and ML across the bariatric surgery pathway, from preoperative planning to postoperative follow-up.

**Methods:**

A systematic search of PubMed and Embase (last accessed August 28, 2025) identified original studies applying AI/ML in MBS. Inclusion criteria were studies involving bariatric patients and AI/ML-based models for clinical or perioperative purposes. Data on study design, sample size, surgical procedure, AI/ML technique, and primary outcomes were extracted. Studies were categorized into preoperative, intraoperative, and postoperative phases.

**Results:**

Of 142 records screened, 27 studies met inclusion criteria. Preoperative applications focused on patient selection, anatomical prediction, and risk stratification, including models predicting weight-loss success, gastroesophageal reflux disease, and hiatal hernia. Intraoperative research explored operative time forecasting, workflow optimization, and automated video analysis for skill assessment and step recognition. Postoperative models addressed complication prediction, nutritional surveillance, and long-term weight trajectory forecasting. Reported accuracy was high (AUC up to 0.93), but external validation and fairness audits were limited. Emerging evidence also supports AI-driven educational tools, including large language models for surgical training.

**Conclusions:**

AI is rapidly transforming bariatric surgery across the preoperative, intraoperative, and postoperative pathway, offering unprecedented opportunities for personalization, efficiency, and quality improvement. From risk prediction to skill assessment and long-term outcome forecasting, its applications can augment, rather than replace, human expertise. Ethical safeguards, transparency, and equitable access remain critical for safe and effective integration.

## Introduction

Recent evidence underscores the rapid expansion of AI in clinical medicine. An overview of systematic reviews (SRs) screened multiple databases and synthesized 161 SRs published up to December 2024, with oncology as the leading field (13.9%) and diagnosis as the predominant aim (44.4%). The authors proposed CLASMODAI (Classification Model for Artificial Intelligence in Clinical Research Reporting) to standardize reporting by input, model, training data, and performance metrics, highlighting the need for methodological transparency [1].

Metabolic and bariatric surgery (MBS) is the most effective intervention for severe obesity [2]. Artificial intelligence (AI) is increasingly applied in bariatric surgery, offering tools for predicting postoperative complications and optimizing weight‑loss outcomes. Machine learning (ML) and deep learning algorithms have achieved predictive accuracies of up to 98% in identifying clinical endpoints using large real‑world datasets. These technologies can enhance surgical decision‑making, improve intraoperative safety, and support personalized treatment strategies. However, their clinical integration requires external validation and methodological standardization to ensure reliability [3, 4]. Aim of our review was to gather and narratively synthetize published evidence on the use of AI in MBS.

## Materials and methods

This narrative review was conducted and reported in line with PRISMA 2020 concepts (checklists and flow diagram templates provided by the PRISMA -Preferred Reporting Items for Systematic Reviews and Meta-Analyses - Executive; figure wording follows PRISMA 2020) [5–6].

A comprehensive search was performed in PubMed and Embase using (((bariatric surgery[MeSH Terms]) AND (machine learning[MeSH Terms]))) AND (artificial intelligence[MeSH Terms]). Databases were accessed on August 28th 2025. Titles and abstracts were screened for eligibility. Inclusion criteria were original research articles investigating AI or ML in patients undergoing bariatric/metabolic surgery. Exclusion reasons were reviews, studies not involving bariatric patients, studies not applying AI/ML in clinical/perioperative contexts. For each included study, we extracted author, year, design, sample size, procedure, AI/ML method, primary outcome, and main findings.

Articles were furtherly grouped by the primary clinical phase: preoperative prediction/planning, intraoperative performance/efficiency, and postoperative outcomes/complications.

## Results

The systematic literature search yielded a total of 142 records (MEDLINE: *n* = 45; EMBASE: *n* = 97). Following the removal of 11 duplicate records, 131 records were screened.

**Fig. 1:**
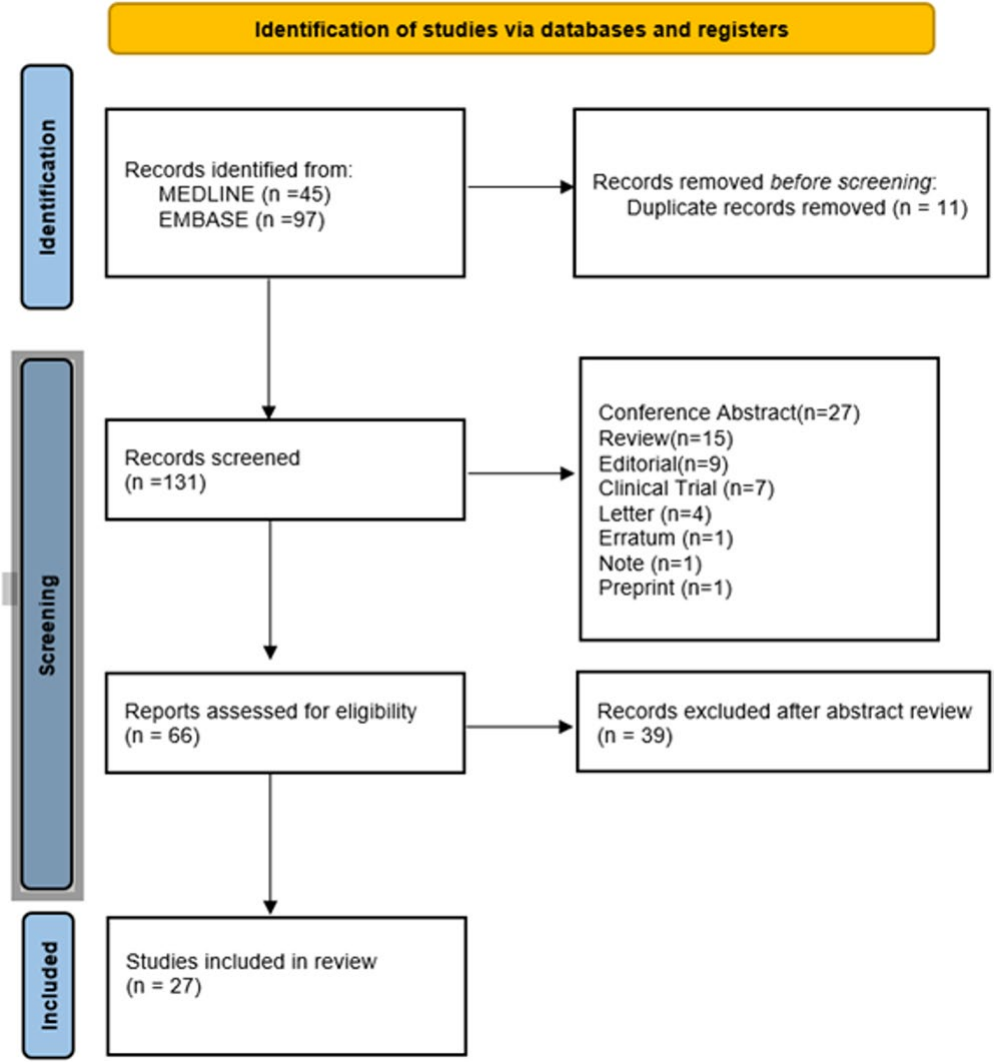
PRISMA 2020 flow diagram

During the identification phase, records were excluded based on publication type and a total of 66 full-text articles were assessed for eligibility. After abstract screening, 39 records were excluded and ultimately, 27 studies met the inclusion criteria and were included in the final review. The selection process is summarized in the PRISMA 2020 flow diagram (Fig. 1).

### Preoperative prediction and surgical planning

Artificial intelligence and machine learning have been extensively applied to optimize preoperative decision‑making and surgical planning in bariatric surgery. These approaches aim to refine patient selection and personalize operative strategies by leveraging complex datasets.

One study developed a clustering‑plus‑XGBoost model to estimate the total length of the small intestine, a critical parameter for tailoring surgical procedures [7]. This model enabled individualized surgical planning by predicting anatomical variability that is otherwise difficult to assess preoperatively. Another investigation employed supervised learning techniques to predict one‑year weight‑loss success following sleeve gastrectomy [8]. The analysis identified biochemical and psychological features as key predictors, highlighting the multifactorial nature of postoperative outcomes and the potential for AI to inform patient counseling. Reproductive health considerations were addressed through a decision‑tree model designed to forecast the likelihood of pregnancy within 12 months after MBS [9]. This predictive tool supports clinicians in providing evidence‑based guidance on family planning and timing of conception. Behavioral outcomes have also been explored. A lifestyle‑based machine learning model was developed to predict the onset of binge‑eating disorder two years after sleeve gastrectomy [10], underscoring the importance of psychosocial factors in long‑term success. Preoperative imaging data have been integrated into AI‑driven workflows as well. A decision‑tree analysis of abdominal ultrasound findings was used to triage patients requiring additional diagnostic workup or concomitant procedures, thereby improving operative planning and resource utilization [11]. Economic forecasting has emerged as another application area. A random‑forest model was constructed to estimate hospital costs associated with bariatric surgery [12], offering a data‑driven approach to financial planning and cost containment. Diagnostic accuracy has also benefited from AI integration. A connectome‑based predictive model utilized neuroimaging data to anticipate six‑month weight‑loss response, suggesting a neurobiological basis for surgical outcomes [13]. A machine learning classifier enhanced the preoperative detection of hiatal hernia [14], while a study developed and validated an AI‑based model to predict the onset of gastroesophageal reflux disease (GERD) after SG using a prospectively maintained dataset of 441 patients. The ensemble model achieved an AUC (area under the curve) of 0.93, with age, weight, preoperative GERD, bougie size, and distance from the pylorus identified as top predictors. The model also provided cutoff values for modifiable surgical parameters to guide decision‑making and reduce GERD risk—highlighting AI’s potential to support preoperative planning and personalized strategies [15] (Table 1).

**Table 1:** Summary of included studies applying machine learning (ML) in the preoperative assessment of bariatric surgery

Publication	Study design	Sample size	Methods (brief)	Main results
Fernando Trebolle et al., 2025	Multicenter RCT	150 post-LSG patients	Dietitian follow-up vs. standard care	Dietitian → ↑%EWL and QoL
Casas Domínguez et al., 2025	Prospective study	82 LSG patients	Predictors of 2-year weight loss	Diet adherence + physical activity → ↑%EWL
Moradi et al., 2025	Nested case–control	473 post-MBS pregnancies	21 variables → ML to predict pregnancy < 12 months	C5.0 AUC 0.92; 13 key factors
Mousavi et al., 2025	Cross-sectional, 2 years post-LSG	450 patients	Lifestyle score + ML for BED	High score → ×3 BED risk
Hany et al., 2024	Studio retrospettivo multicentrico	4.418 pazienti MBS	Ecografia addominale preop. + ML per classificare reperti	15,9% benefici da diagnosi colecistite; 1,4% interventi rinviati; DT AUC 0,976
Ochs et al., 2023	Retrospective single-center	602 MBS patients	14 clinical/surgical variables → 6 ML models to predict hospital cost	RF MAPE 12.7%; top: anesthesia time, LOS, surgery time
Zhang et al., 2021	Prospective fMRI study	37 LSG patients	Baseline RSFC + ML (Siamese-KNN) to predict optimal 6-month weight loss	Acc. 83.8%, AUC 0.84; stronger fronto-parietal connectivity in responders
Assaf et al., 2022	Retrospective study	2,482 MBS patients	3 ML models to improve preop hiatal hernia diagnosis vs. contrast swallow	Sensitivity ↑ from 38.5% to 60.2%; specificity ~ 93%
Emile et al., 2022	Retrospective study	441 SG patients	AI ensemble model to predict GERD after SG from clinical/technical data	AUC 0.93; top predictors: age, weight, preop GERD, bougie < 38 Fr, < 3 cm from pylorus

*Legend*: *LSG  *Laparoscopic Sleeve Gastrectomy, *MBS *Metabolic and Bariatric Surgery, *%EWL* Percentage of Excess Weight Loss, *QoL*  Quality of Life, *ML*  Machine Learning, *BED*  Binge Eating Disorder, *RF* Random Forest, *RSFC * Resting-State Functional Connectivity,*KNN * K-Nearest Neighbors, *GERD*  Gastroesophageal Reflux Disease,* SG * Sleeve Gastrectomy, *AUC*  Area Under the Curve, *MAPE*  Mean Absolute Percentage Error, *DT* Decision Tree, *Fr * French (bougie size)

###  Intraoperative performance and efficiency

Intraoperative applications of AI have primarily focused on predicting operative duration and assessing technical performance. Building on this, an Extreme Gradient Boosting (XGBoost) model trained on MBSAQIP (Metabolic and Bariatric Surgery Accreditation and Quality Improvement Program) data demonstrated superior accuracy compared to linear models in predicting operative time, with procedure type and surgical approach emerging as dominant predictors [16]. These insights can inform scheduling, resource allocation, and workflow optimization. Skill assessment represents another promising domain. Performance analytics leveraging synthetic data augmentation were employed to classify proficiency levels in endoscopic sleeve gastroplasty (ESG) [17], illustrating the potential of AI to support competency‑based training and continuous quality improvement. A convolutional AI model automatically labeled the steps of Roux-en-Y gastric bypass (RYGB) across 545 surgical videos. Trained 390 and tested on 60 videos, it achieved F1 scores > 90% for 7/12 steps and outperformed trainee annotators in several categories. The system was comparable to manual annotation and showed potential to improve surgical training by assessing learning curves and identifying procedural landmarks at scale [18] (Table 2).

**Table 2:** Studies applying machine learning (ML) for surgical training, performance assessment, intraoperative analysis, and operative time prediction in bariatric and metabolic surgery

Publication	Study design	Sample size	Methods	Key results
Dials et al., 2023	Simulation	7 ESG cases (4 experts, 3 novices)	VR-ESG tasks; SMOTE + PCA; 6 ML models	AI distinguished experts vs. novices with 100% accuracy; main predictors: suture quality, completion time
Fer et al., 2023	Video analysis	545 RYGB videos	R(2 + 1)D + MS-TCN vs. surgeon annotations	AI matched or exceeded surgeons in several steps
Kang et al., 2024	Retrospective	668 723 MBS cases	Regression, RF, SVM, GBT, XGBoost; SHAP analysis	XGBoost achieved the highest accuracy, predicting operative time with an average error of about 40 min (R² = 0.31). The most influential factors were surgery type, surgical approach, concurrent procedures, patient age, BMI, and comorbidities.

*Legend*: *ESG* Endoscopic Sleeve Gastroplasty, *VR*  Virtual Reality, *SMOTE*  Synthetic Minority Oversampling Technique,* PCA* Principal Component Analysis, *ML*  Machine Learning, *SVM*  Support Vector Machine, *RYGB*  Roux-en-Y Gastric Bypass, R(2 + 1)D  Residual (2 + 1)-Dimensional Convolutional Network, *MS-TCN * Multi-Stage Temporal Convolutional Network F1 = F1-score, *MAE*  Mean Absolute Error, *RMSE*  Root Mean Square Error, *MBS* Metabolic and Bariatric Surgery, *GBT*  Gradient-Boosted Tree, *RF* Random Forest, *BMI*  Body Mass Index, *GERD*  Gastroesophageal Reflux Disease

### Postoperative outcomes and complications

Postoperative research has concentrated on predicting complications, monitoring nutritional status, and modeling long‑term weight trajectories. Psychological and lifestyle determinants of weight regain were analyzed using machine learning approaches [19], enabling early identification of high‑risk patients. A prospective study integrated mobile application alerts and symptom tracking with clinical variables to enhance early detection of postoperative complications, outperforming models based solely on preoperative data [20]. Similarly, a run‑to‑run autoregressive model utilized continuous glucose monitoring data to forecast post‑bariatric hypoglycemia under real‑world conditions [21]. Translational studies have applied AI to molecular data, identifying hepatic hub genes such as PPARA and CPT1A that mediate metabolic remodeling after surgery [22]. In parallel, nonlinear and explainable models surpassed traditional scoring systems in predicting 30‑day complications [23], while fairness audits were incorporated into MBSAQIP‑based models for serious adverse events, addressing concerns of algorithmic bias [24]. Longitudinal weight prediction tools have also been developed. A single‑center pilot introduced a web‑based platform for forecasting BMI across multiple time points up to five years [25], while nutritional surveillance has been enhanced through AI‑driven models that identified predictors of recurrent thiamine deficiency and gaps in vitamin B1 monitoring [26], as well as patterns associated with underrecognized vitamin C deficiency [27]. An internationally validated calculator has been developed to interpretate five‑year weight trajectories for shared decision‑making [28]. Additional models targeted specific adverse events, including gastrointestinal bleeding [29] and major adverse cardiac events [30], while ensemble learning techniques such as Super Learner were applied to estimate 30‑day readmission risk [31]. Further studies explored predictors of ESG success [32] and biomarker screening for liver function post‑surgery [33] (Table 3).

**Table 3:** Machine learning (ML) and data-driven approaches in bariatric and metabolic surgeryfor predicting outcomes, complications, surgical performance, and mechanistic insights

Study	Design	Sample	Methods	Main findings
Vieira etal., 2025	Cross-sectional,single-center	124 post-bariatricpatients	Validatedquestionnaires;Poisson regression; ML (RF, XGBoost)	Psychological factorsimprove prediction of weight regain; XGBoost perform best.
Farinellaet al.2025	Prospective,single-center	104 bariatricpatients	Pre/post-opmodels; PCA,SMOTE	Dynamic monitoringimproves predictionover static data.
Prendin etal., 2025	Prospectiveobservational	47 patients withPBH post-RYGB	CGM data; rAR,ARIMA, NN, LSTM,CNN-LSTM, RF,LGB	rAR predicts PBH withbalanced F1, precision,and recall.
Li et al.,2025	Multi-dataset +experimental validation	131 humansamples + 40mice	DEG/CPG analysis;99 MLcombinations;qRT-PCR	Six hub genesdistinguish pre/postMBS; RYGB normalizesgene expression.
Zucchiniet al.2024	Retrospective,single-center	424 MBS patients	LR. SVM, RF, KNN,MLP, XGB; SHAP	RF outperformsMBSAQIP (AUROC 0.88vs 0.64); keypredictors: ferritin,triglycerides, ALT, GGT.
Kang etal., 2024	Retrospective,MBSAQIP 2016-2020	40,858 patients(afterundersampling)	LR, RF, GBT, XGB:fairness metrics	XGB highest AUROC; RFbest recall; LR mostequitable.
Ochs etal., 2024	Retrospective,single-center	1,104 bariatricpatients	RF, XGB, MLP.CNN, RNN,Transformer,SVM, AdaBoost	RF best short-term; MLPbest at 5 years;predictors: BMI, age,comorbidities.
Parrot etal., 2024	Prospective,single-center	97 patients(RYGB or SG)	Body composition(BIA, DXA); ML(RF, XGB, SVM)	RF predicts ASM withhighest accuracy;useful for nutritionalassessment.
Parrott etal., 2023	Retrospective,multicenter	5,946 MBSpatients (187with VCmeasured)	ML (BayesNet,RF); 52 labparameters	ML predicts vitamin Cdeficiency (VCD) withAUC ~0.70; VCD linkedto other deficiencies.
Saux etal., 2023(SOPHIA)	Retrospective,multinational	10,231 patientsfrom 12 centersin 10 countries	LASSO + CART;externalvalidation on 10cohorts and 2RCTs	ML predicts 5-year BMIwith RMSE = 4.7 kg/m2;key variables: age,weight, height,procedure type,diabetes, smoking.
Hsu et al.,2023	Retrospective,MBSAQIP 2020	159,959 bariatricpatients	ML (RF, XGB, NN)VS LR; SHAP	RF predicts Gl bleedingpost-op with AUROC =0.764 vs LR = 0.709top features: proceduretype, age, duration,hematocrit, creatinine.
Romero-Velez etal., 2023	Cross-sectional,MBSAQIP 2015-2019	755,506 bariatric patients	ML (NN, XGBoost)VS LR; AUCcomparison	NN predicts MACE withAUROC = 0.798,outperforming LR(0.790) and XGBoost(0.787).
Torquatiet al.2022	Retrospective,MBSAQIP 2015–2018	393,833 bariatricpatients	ML (Super Learnerensemble) vs LR;5-fold CV, NRI, IDI	Super Learner predicts30-day readmissionwith AUROC = 0.674 vsLR = 0.650; betterreclassification anddiscrimination.
Vannucciet al.,2023	Multicenter,ESG 2016-2021	404 ESG patients	ML (LR, SVM, RFC,KNN); internal,external,temporalvalidation	ML predicts ESGsuccess (TWL ≥10%,EWL ≥25%) with AUCup to 0.88 using follo w-up data.
Jiang etal., 2023	Bioinformatics+ cellvalidation	74 liver samplespre/post-BS	WGCNA + SVM-RFE + GSEA + OilRed O + WB	Four biomarkers (SRGN,THEMIS2, SGK1, FPR3)linked to lipidmetabolism andimmune activationpost-BS; SRGN silencingreduces lipogenesis inHepG2 cells.

*ML* Machine Learning, *RF* Random Forest, *XGBoost *eXtreme Gradient Boosting, *LR *Logistic Regression, *SVM *Support Vector Machine, *KNN *K-Nearest Neighbors, *MLP *Multi-Layer Perceptron, *CNN *Convolutional Neural Network, *RNN *Recurrent Neural Network, *SHAP *SHapley Additive exPlanations, *SMOTE *Synthetic Minority Over-sampling Technique, *PCA *Principal Component Analysis, *CGM *Continuous Glucose Monitoring, *PBH *Postprandial Hyperinsulinemic Hypoglycemia, *RYGB *Roux-en-YGastric Bypass, *SG *Sleeve Gastrectomy, *MBS *Metabolic and Bariatric Surgery, *MBSAQIP *Metabolic and Bariatric Surgery Accreditation and Quality Improvement Program, *VC *Vitamin C, *VCD *Vitamin CDeficiency, *ASM *Appendicular Skeletal Muscle, *ESG *Endoscopic Sleeve Gastroplasty, *TWL *Total WeightLoss, *EWL *Excess Weight Loss, *WGCNA *Weighted Gene Co-expression Network Analysis,* SVM-RFE* Support Vector Machine - Recursive Feature Elimination, *GSEA *Gene Set Enrichment Analysis, *WB *Western Blot, *MACE *Major Adverse Cardiac Events, *AUC *Area Under the Curve, *RMSE *Root Mean SquareError, *NRI *Net Reclassification Index, *IDI *Integrated Discrimination Improvement, *RCT *RandomizedControlled Trial

## Discussion

A recent international consensus provides a structured framework for responsible AI adoption in MBS. In a Delphi involving 68 experts from 35 countries, agreement was reached on AI’s potential to enhance surgical training, decision‑making, patient selection, cost‑effectiveness, and outcome prediction, while emphasizing ethical safeguards and informed consent. The panel supported integrating AI into surgical curricula and highlighted the promise of AI‑driven robotics and genomics for personalized care. Collectively, these statements outline principles for safe, effective, and ethically grounded AI deployment in surgical practice [34].

Beyond predictive analytics and perioperative optimization, recent studies have explored the educational potential of large language models (LLMs) in surgical training. Lee et al. conducted a comparative analysis of ChatGPT‑4, Bing, and Bard using 200 multiple‑choice questions from the ASMBS (American Society for Metabolic and Bariatric Surgery) Textbook of Bariatric Surgery. ChatGPT‑4 achieved the highest accuracy (83.0%), outperforming Bard (76.0%) and Bing (65.0%), particularly in treatment/procedure and complication domains. Notably, ChatGPT‑4 answered all questions, demonstrating superior consistency across question types. These findings suggest that advanced LLMs could serve as valuable adjuncts in bariatric surgery education, supporting knowledge acquisition and reinforcing clinical decision‑making [35].

Similarly, Kothari et al. evaluated ChatGPT‑4’s performance on the American Board of Surgery In‑Training Examination (ABSITE), comparing its accuracy to that of surgical residents. ChatGPT‑4 achieved an overall accuracy of 76.5%, comparable to postgraduate year‑1 residents and markedly superior to earlier AI models. Performance was strongest in trauma, critical care, and gastrointestinal surgery, though variability persisted across subspecialties. These results underscore the potential role of LLMs as supplementary educational tools, complementing traditional learning methods and potentially enhancing surgical curricula [36].

Our review highlighted the broad potential of AI to enhance decision-making, proficiency, and patient outcomes across all phases of bariatric surgery. However, the heterogeneity of models, limited external validation, and risk of algorithmic bias underscore the need for cautious interpretation.

AI-driven early and accurate preoperative identification of hiatal hernia (HH) [14] and prediction of postoperative gastroesophageal reflux disease (GERD) risk [15] may substantially influence surgical strategy in bariatric patients. Detecting HH before surgery enables timely repair, potentially reducing postoperative reflux and improving long‑term outcomes. Similarly, anticipating GERD risk through AI‑based models allows for tailored operative planning, including adjustment of modifiable technical parameters. Such proactive assessment supports personalized care, optimizes patient counseling, and may decrease the incidence of postoperative complications. AI-driven tools can enhance surgical training by objectively assessing technical skills, classifying proficiency levels, and tracking learning curves [18, 19]. Automated video analysis and step recognition enable large-scale, consistent feedback, reducing reliance on time-intensive manual annotation. Accurately predicting complications such as postoperative gastrointestinal bleeding [30] enables surgeons to stratify risk, tailor perioperative management, and implement targeted preventive strategies. Likewise, forecasting micronutrient deficiencies [27, 28] supports proactive nutritional follow-up, ensuring timely supplementation and reducing long-term morbidity. Furthermore, tools like the SOPHIA (Stratification of Obesity Phenotypes to Optimize Future Therapy calculator) [29] and similar platforms can provide patients in the preoperative phase with credible, individualized projections of weight loss and diabetes remission, enhancing shared decision-making and setting realistic expectations.

However, ethical concerns—particularly regarding data privacy, transparency, and equitable access—must be addressed to ensure responsible adoption. Ultimately, AI should assist, not replace, clinical expertise, with robust human oversight remaining essential for safe and ethical integration into daily surgical practice.

Indeed, surgeons will not be replaced by AI, but by those who harness its capabilities to deliver precision medicine. The integration of AI into surgical practice empowers clinicians to make data-driven, patient-specific decisions, enhancing both safety and outcomes. Those who adapt will lead the evolution toward more personalized, efficient, and effective care.

## Strengths and limitations of this review

Strengths include a focused clinical scope and PRISMA‑concordant reporting. A formal risk of bias assessment tool could not be uniformly applied due to methodological heterogeneity and incomplete reporting across AI/ML studies, key sources of bias were qualitatively assessed and discussed throughout the manuscript.

Moreover, substantial heterogeneity across the included studies limited the feasibility of quantitative synthesis. The AI/ML models varied widely in their methodological approaches, ranging from traditional supervised algorithms to deep‑learning architectures, each with different training strategies and performance characteristics. Dataset size and quality also differed considerably, with some studies relying on small single‑center cohorts and others using large administrative registries, affecting generalizability. Reporting performance metrics and validation methods was inconsistent, with several studies lacking external validation despite reporting high accuracy, including AUC values up to 0.93. Clinical endpoints were equally diverse, spanning weight‑loss outcomes, postoperative complications, nutritional deficiencies, operative time, and psychological variables. This variability reflects the broad applicability of AI in MBS but complicates direct comparison and clinical translation. As noted in the manuscript, the heterogeneity of models, limited external validation, and potential algorithmic bias underscore the need for cautious interpretation and highlight the importance of standardized reporting and multicenter validation in future research.

## Conclusion

AI is rapidly transforming bariatric surgery across the preoperative, intraoperative, and postoperative pathway, offering unprecedented opportunities for personalization, efficiency, and quality improvement. From risk prediction to skill assessment and long-term outcome forecasting, its applications can augment, rather than replace, human expertise. Ethical safeguards, transparency, and equitable access remain critical. In the field of MBS, safer, more precise, and patient‑centered surgical care will likely be achieved through the synergistic collaboration of skilled surgeons and advanced AI technologies.

## Data Availability

No datasets were generated or analysed during the current study.
